# The Impact of Bronchodilator Therapy on Systolic Heart Failure with Concomitant Mild to Moderate COPD

**DOI:** 10.3390/diseases6010004

**Published:** 2017-12-28

**Authors:** Mahoto Kato, Kazuo Komamura, Masafumi Kitakaze, Atsushi Hirayama

**Affiliations:** 1 Division of Cardiology, Department of Medicine, Nihon University School of Medicine, 30-1 Ohyaguchi Kamicho, Itabashi, Tokyo 173-8610, Japan; hirayama.atsushi@nihon-u.ac.jp; 2 Division of Medical Technology Faculty of Nutrition, Kobe Gakuin University, Kobe 651-2180, Japan; komamura@nutr.kobegakuin.ac.jp; 3 Department of Clinical Medicine and Development, and Department of Cardiovascular Medicine, National Cerebral and Cardiovascular Centre, Osaka 565-0873, Japan; kitakaze@zf6.so-net.ne.jp

**Keywords:** heart failure with reduced ejection fraction, chronic obstructive pulmonary disease, tiotropium, anticholinergic bronchodilator

## Abstract

In older adults, chronic obstructive pulmonary disease (COPD) is commonly associated with heart failure with reduced ejection fraction (HFrEF), and the high prevalence of this combination suggests that customized treatment is highly necessary in patients with COPD and HFrEF. To investigate whether the treatment of COPD with tiotropium, an anticholinergic bronchodilator, reduces the severity of heart failure in patients with HFrEF complicated by mild to moderate COPD, forty consecutive participants were randomly divided into two groups and enrolled in a crossover design study. Group A inhaled 18 μg tiotropium daily for 28 days and underwent observation for another 28 days. Group B completed the 28-day observation period first and then received tiotropium inhalation therapy for 28 days. Pulmonary and cardiac functions were measured on days 1, 29, and 56. In both groups, 28 days of tiotropium inhalation therapy substantially improved the left ventricular ejection fraction (from 36.3 ± 2.4% to 41.8 ± 5.9%, *p* < 0.01, in group A; from 35.7 ± 3.8% to 41.6 ± 3.8%, *p* < 0.01, in group B) and plasma brain natriuretic peptide levels (from 374 ± 94 to 263 ± 92 pg/mL, *p* < 0.01, in group A; from 358 ± 110 to 246 ± 101 pg/mL, *p* < 0.01, in group B). Tiotropium inhalation therapy improves pulmonary function as well as cardiac function, and reduces the severity of heart failure in patients with compensated HFrEF with concomitant mild to moderate COPD.

## 1. Introduction

It has been reported that pharmacological treatment related to chronic heart failure (CHF), such as beta-adrenergic receptor blockers (beta-blockers) and angiotensin-converting enzyme inhibitors (ACE-I), not only alleviates symptoms but also improves prognosis, particularly through the suppression of neurohormonal factors. However, in older patients with deteriorated and severe CHF, exercise tolerance is limited by the symptoms, and the rehospitalization rate due to acute exacerbation and the mortality rate are still high even with the currently available optimal treatment [1,2,3]. Therefore, it is necessary to attempt to treat each patient with CHF individually rather than providing uniform treatment.

The number of older adults with coexisting chronic obstructive pulmonary disease (COPD) and heart failure with reduced ejection fraction (HFrEF) has recently reached global epidemic levels, with each of the diseases affecting >10 million patients. The reported prevalence of COPD in patients with HFrEF ranges widely, from 11% to 52% in North America and from 9% to 41% in Europe [4,5]. These two common diseases exert adverse effects on one another, reduce the patient health-related quality of life (HRQoL) and exercise tolerance, and considerably increase morbidity and mortality. We focused on this unique but large population and attempted to find treatments that could overcome the limitations of existing CHF therapies.

Tiotropium is a once-daily, long-acting inhaled anticholinergic drug that provides improvements in airflow and hyperinflation for at least 24 h, thus reducing dyspnea and improving exercise tolerance and HRQoL [6,7]. Therefore, it is a commonly prescribed therapeutic agent for patients with COPD. Recently, inhalation of tiotropium has also been reported to suppress the occurrence of congestive heart failure and the onset of myocardial infarction, and to reduce the risk of cardiovascular events and mortality [8,9]. Furthermore, tiotropium was reported to improve COPD without exacerbating the coexisting CHF, as reflected in the decrease in both the brain natriuretic peptide (BNP) and serum norepinephrine (NE) levels [10]. On the basis of these previous reports, we investigated in the present study whether anticholinergic bronchodilator therapy reduces the severity of both COPD and heart failure.

## 2. Patients and Methods

### 2.1. Participants

The inclusion criteria for this study were as follows: (1) stable COPD, confirmed with spirometry as defined by the Global Initiative for Chronic Obstructive Lung Disease (GOLD) Scientific Committee [11]; (2) chronic left-sided systolic heart failure of New York Heart Association (NYHA) functional class I or II, as defined by the American College of Cardiology/American Heart Association guidelines [12]; (3) reduced left ventricular (LV) ejection fraction (LVEF) of <40%; and (4) high level of serum BNP (>100 pg/mL) with optimal medical therapy for at least three months before enrollment. The patients had already taken oral diuretics, ACE-I, angiotensin receptor blockers (ARBs), calcium-channel blockers (CCBs), and beta-blockers. Their previous medication remained unchanged throughout the study period.

### 2.2. Study Protocol

**Figure 1:**
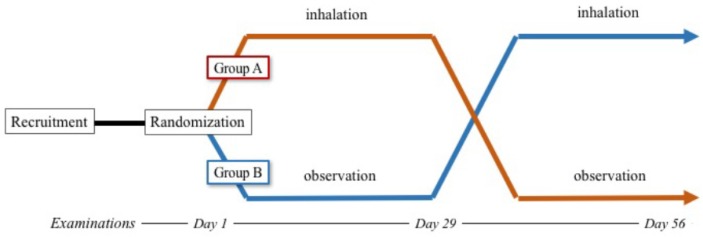
Study protocol.

The enrolled participants were randomly divided into two groups, A and B, following a 2-week baseline period. The participants inhaled 18 μg tiotropium once daily between 6:00 a.m. and 8:00 a.m. through a dry powder inhaler device (HandiHaler^®^; Boehringer Ingelheim, Ingelheim am Rhein, Germany) [13]. Inhalation of tiotropium took place after the patients were given adequate instruction and training by professional nurses. Continuation of inhalation was confirmed at each outpatient examination. Participants who could not continue the inhalation therapy were excluded from this clinical trial. The participants in group A received tiotropium once a day for 28 days and underwent observation for another 28 days. The participants in group B were observed without inhalation for 28 days and then received tiotropium for 28 days (Figure 1). During these protocols, the other medications taken by the participants were unchanged. We measured the pulse oximetry oxygen saturation (SpO_2_), pulmonary function, 6-min walk distance (6MWD), echocardiographic parameters, and laboratory markers on days 1, 29, and 56. The study protocol was approved by an independent ethics committee, and written informed consent was obtained from all patients.

#### 2.2.1. Pulmonary Function

We evaluated pulmonary function by using spirometry, which was conducted between 9:00 a.m. and 11:00 a.m. in accordance with American Thoracic Society (ATS) standards. The predicted normal values for forced expiratory volume (FEV) and forced vital capacity (FVC) were derived from standard equations [14].

#### 2.2.2. Six-Minute Walk Distance

We measured the distance the patient was able to walk, self-paced, on a flat, hard surface in a period of 6 min. This test was conducted between 9:00 a.m. and 11:00 a.m., per ATS guidelines [15].

#### 2.2.3. Echocardiography

Echocardiographic examination was performed by skilled echocardiographers who were blinded to the purpose of the study. The echocardiographic parameters of LV systolic and diastolic function were obtained. The conventional parameters of LV dimension and LVEF were also obtained and measured using a modified Simpson method.

#### 2.2.4. Laboratory Measurements

During a 30-min supine rest, blood samples for biochemistry and complete blood count were drawn from patients who had fasted since 7:00 p.m. the previous night. Plasma BNP and NE levels were measured with a specific immunoradiometric assay using commercial kits (Shionogi, Osaka, Japan). Enzyme-linked immunosorbent assay was performed. The normal ranges of BNP and NE with these measurements are ≤18.6 pg/mL and 15–57 pg/mL, respectively.

#### 2.2.5. Statistical Analysis

We presented data as numbers (percentages) for categorical variables and as means ± standard deviations for continuous variables in the tables. Obtained parameters were compared during different conditions with repeated-measures analysis of variance followed by Tukey’s post hoc test to establish the statistical significance of tests. We also tested the relationship between the percentage decrease of BNP and the absolute increase of FEV1.0 by using Pearson’s correlation coefficients. A two-sided *p*-value of <0.05 was considered statistically significant. All analyses were performed using SAS statistical software (version 9.2; SAS Institute Inc., Cary, NC, USA).

## 3. Results

### 3.1. Patients’ Characteristics

Fifty-two consecutive outpatients diagnosed as having COPD complicated by HFrEF met the inclusion criteria and were recruited for this study. Patients with a history of asthma (*n* = 2), allergic rhinitis (*n* = 1), or allergies (*n* = 1); an increased total eosinophil count (*n* = 1); or orthopedic disorder (*n* = 5) were excluded. Patients in GOLD stage III (severe; FEV1.0/FVC < 70% and 30% ≤ FEV1.0(%predict) < 50%) and in GOLD stage IV (very severe; FEV1.0/FVC < 70% and 30% > FEV1.0(%predict)) were not enrolled (*n* = 2).

At the time of enrollment into this study, no patient had clinical evidence of fluid overload. All patients were classified as having NYHA I (*n* = 13) or II (*n* = 27) CHF. Twenty-two patients had ischemic heart disease (55%) and 13 had hypertensive heart disease (32.5%). The mean LVEF, arterial pressure, and plasma BNP level were 36.5 ± 2.0%, 120 ± 7/80 ± 8 mmHg, and 372 ± 105 pg/mL, respectively. The mean FEV1.0 and 6MWD were 1.56 ± 0.18 L and 405 ± 62 m, respectively. Fifteen patients (37.5%) had GOLD stage I/mild COPD (FEV1.0/FVC < 70% and FEV1.0(%predict) ≥ 80%), and 25 patients (62.5%) had GOLD stage II/moderate COPD (FEV1.0/FVC < 70% and 50% ≤ FEV1.0(%predict) < 80%). Twenty-two patients received beta-blockers, all patients received either ACE-I or ARB, three patients received a CCB, and 32 patients received diuretics. After randomization, there was no significant difference between the two groups (Table 1). During the study period, no patient dropped out owing to discontinuation of tiotropium inhalation or any adverse events, and there were no unexpected visits.

### 3.2. Clinical Effects of Tiotropium for Patients with HFrEF and COPD

#### 3.2.1. Blood Pressure

In both groups, blood pressure significantly decreased after the inhalation of tiotropium, although medications were not changed during the study period (Table 2 and Table 3).

#### 3.2.2. Six-Minute Walk Distance

**Figure 2:**
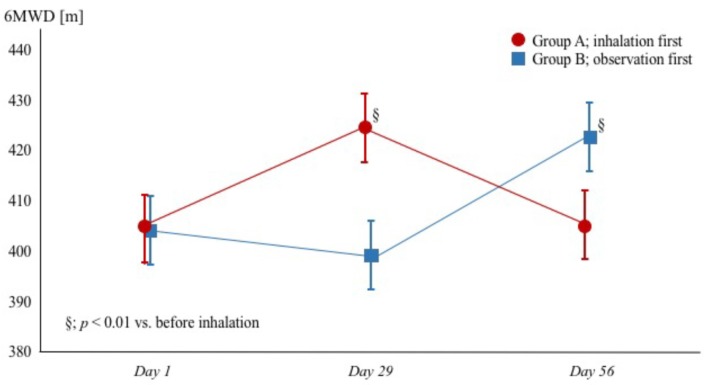
Changes of 6MWD in patients with heart failure with reduced ejection fraction and chronic obstructive pulmonary disease. Red circles (●) and blue squares (▪) represent group A (tiotropium + observation) and group B (observation + tiotropium), respectively. 6MWD = 6-min walk distance.

Treatment with tiotropium inhalation improved the 6MWD from 405 ± 57 to 424 ± 46 m (*p* < 0.01) in group A and from 399 ± 74 to 422 ± 58 m (*p* < 0.01) in group B, and termination of inhalation reduced the distance in group A to 405 ± 63 m (*p* < 0.01) (Figure 2).

#### 3.2.3. Pulmonary Function

FEV1.0 and FVC were elevated by the inhalation of tiotropium in both groups, whereas termination of inhalation therapy decreased FEV1.0 and FVC in group A (Table 2 and Table 3).

#### 3.2.4. Cardiac Function

In both groups, LVEF improved (*p* < 0.01) and diastolic LV diameter (LVDd) increased (*p* < 0.01); in addition, the pressure gradient between the right atrial and right ventricle chamber (PG(RA-RV)) significantly decreased without any change to the diameter of the inferior vena cava (Table 2 and Table 3).

#### 3.2.5. Plasma BNP and NE Levels

**Figure 3:**
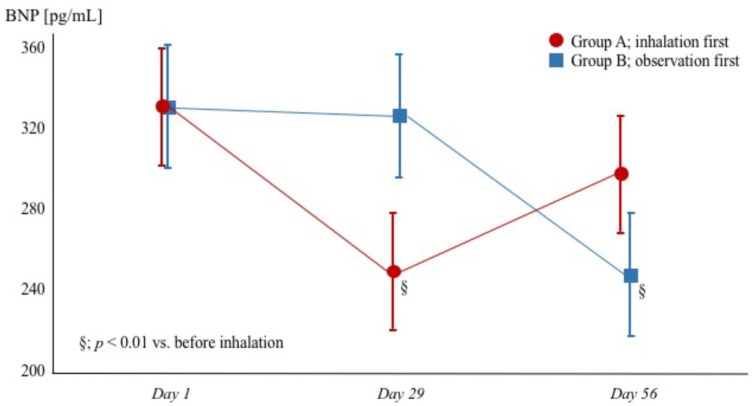
Changes in BNP levels in patients with heart failure with reduced ejection fraction and chronic obstructive pulmonary disease. Red circles (●) and blue squares (▪) represent group A (tiotropium + observation) and group B (observation + tiotropium), respectively. BNP = brain natriuretic peptide.

Although there was no significant hemodynamic change, tiotropium inhalation decreased the plasma BNP and NE levels in both groups. Termination of inhalation therapy increased the plasma BNP and NE levels in group A (Figure 3 and Table 2).

#### 3.2.6. Relation between Change in FEV and LVDd

Tiotropium inhalation increased not only FEV1.0 but also LVDd in both groups; however, the systolic LV diameter did not change (Table 2 and Table 3). Although it was not significant, there was a tendency for the changes in LVDd and FEV1.0 to be related (r = 0.57, *p* = 0.096).

#### 3.2.7. Relationship between Change in FEV and BNP

**Figure 4:**
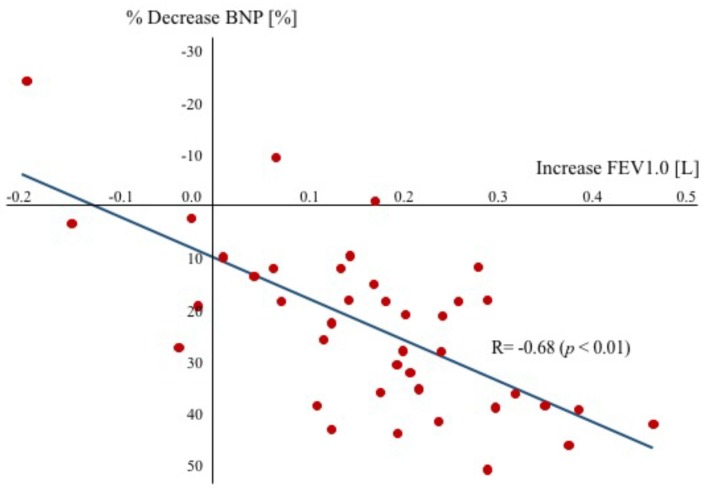
Relationship between the percentage decrease in plasma BNP levels and the absolute increase in FEV1.0. A significant relationship was observed between the percentage decrease in BNP and the increase in FEV1.0. BNP = brain natriuretic peptide, FEV = forced expiratory volume.

In the present study, we demonstrated that anticholinergic bronchodilator therapy decreases the plasma BNP level in patients with HFrEF complicated by COPD, and there was a significant relationship between the percentage decrease of the plasma BNP level and the absolute change of FEV1.0 resulting from tiotropium inhalation (r = −0.68, *p* < 0.01) in both groups (Figure 4). However, there was no relationship between plasma NE levels and FEV1.0.

### 3.3. Adverse Effects

Several adverse events occurred during the study period. Dry mouth occurred in 12 patients, nasal congestion in four patients, nose bleeding in three patients, and constipation in one patient. The proportion of patients who experienced adverse effects during the course of the study period was similar in both groups. The only adverse effect that differed significantly between the tiotropium inhalation and observation groups was dry mouth, which occurred in nine patients (22.5%) in the tiotropium inhalation group and in three patients (7.5%) during the observation period. No adverse events occurred that led to the discontinuation of the study.

## 4. Discussion

In the present study, we demonstrated the clinical effects of tiotropium inhalation therapy for patients with mild to moderate COPD. As in many reports thus far, we found that the treatment improved pulmonary function, particularly increasing FEV1.0 and consequently increasing 6MWD. Furthermore, we demonstrated that tiotropium inhalation therapy increased the LVEF, decreased the PG(RA-RV), and decreased the plasma BNP level, which indicate that the treatment reduced the severity of heart failure.

### 4.1. Possible Mechanisms by Which Anticholinergic Bronchodilator Improves HFrEF

It has been shown that the larger the degree of pulmonary emphysema, the smaller the left ventricle, and the lower the FEV1.0, the smaller the left ventricle, which indicate that hyperinflation of the lungs affects the size of the heart and is associated with an extended disturbance of the heart [16]. In patients with HFrEF, the contraction force of the LV is reduced because the cardiac output is usually maintained by LV dilatation. The excess expiratory load in patients with COPD increases the intrathoracic pressure and pushes the right ventricle, resulting in reduced gradient for venous return [17]. In addition, the marked hyperinflation associated with COPD may reduce the speed and volume of LV filling because of competition for intrathoracic space [18]. Thus, the effect of decreased cardiac output is remarkable in patients with HFrEF. The primary mechanism for improvement likely involves reduced obstruction, improved gradient for venous return, and a mild deflation, thereby improving cardiac filling pressures. Indeed, a prior article reported that tiotropium inhalation improved diastolic function and reduced LV end-diastolic pressure, as assessed with tissue Doppler echocardiography [10].

Skeletal muscle alterations in patients with COPD include decreased muscle strength and mass, with reduced cross-sectional area, fiber shift with atrophy of type I oxidative fibers, and increase in glycolytic type IIa and IIb fibers accompanied by an increase in glycolytic activities and a decrease in oxidative enzymatic activities [19,20]. Muscle atrophy contributes to respiratory muscle fatigue, which causes patients with CHF to discontinue exercising before they have exhausted their cardiac reserves [21]. Furthermore, respiratory muscle weakness increases neurohormonal activities. Thus, improvement of air trapping resulting from inhalation of tiotropium decreases the inspiratory load and results in reduced fatigue on inspiration and improved exercise capacity.

We investigated the changes in SpO_2_ and found that tiotropium inhalation therapy improved oxygen delivery to the organs and muscles of the body at rest, which indicates that bronchodilator therapy improves the speed of oxygen delivery to the skeletal muscles during exercise. Consequently, increased oxygen delivery might suppress neurohormonal activities.

### 4.2. Sustained Effects after Termination of Tiotropium

After the termination of tiotropium inhalation, the PG(RA-RV) and plasma NE levels remained suppressed. Because the drug effects of tiotropium are clearly disappearing after the termination of inhalation therapy, these phenomena must be explained by the fact that the pathology of heart failure remains stable. Thus, as pulmonary function and hemodynamics improved during the administration of tiotropium therapy, the promotion of neurohormonal factors and inflammation are alleviated, and this good condition is sustained. However, it is unclear how long the beneficial effect lasts after termination, and it is presumed that it will eventually return to the state before administration. In fact, these data were slightly worse than those during tiotropium inhalation (Table 2 and Table 3).

### 4.3. Safety of Tiotropium in Patients with HFrEF

We observed no adverse effect of tiotropium inhalation therapy other than dry mouth in the present study. Beta-stimulant as a bronchodilator was reported to activate the neurohormonal system and to exacerbate CHF [22]. However, tiotropium use was not associated with any cardiac safety concerns, as defined by electrocardiographic evaluations in placebo-controlled clinical trials [23]. Furthermore, tiotropium was reported to suppress the activation of the sympathetic nervous system [24] and to not increase the risk of cardiac events [22,25]. Indeed, we observed that tiotropium inhalation therapy resulted in no adverse events, such as tachycardia, atrial fibrillation, or exacerbation of CHF.

### 4.4. Study Limitations

In this study, we investigated patients with compensated heart failure with a reduced LVEF of <40%; no data exist on heart failure with a preserved LVEF of >40%. Although we investigated patients with mild to moderate ambulatory COPD, no data exist on HFrEF complicated by severe or very severe COPD. Because the study period was only two months long, whether inhalation of tiotropium influences mortality in this population is unknown. Clinical trials are needed to investigate the long-term prognosis on larger and more varied populations with multicenter implementation.

### 4.5. Clinical Implications

Dyspnea and loss of exercise capacity are symptoms of both COPD and HFrEF, and cause a reduction in HRQoL. When administrated to patients with COPD, tiotropium proved to improve cardiac function and reduce the severity of heart failure, as well as improved pulmonary function, resulting in improved HRQoL in patients with compensated HFrEF complicated by mild to moderate COPD. Although the total management in this specific but large population is still controversial, anticholinergic inhalation therapy is suggested as a potentially effective therapy.

## 5. Conclusions

In the present study, we demonstrated that anticholinergic bronchodilator therapy not only improved pulmonary function but also reduced the severity of heart failure in patients with compensated HFrEF complicated by mild to moderate COPD.

## Figures and Tables

**Table 1:** Patient characteristics.

	All (*n* = 40)	Group A (Tiotropium + Observation; *n* = 20)	Group B (Observation + Tiotropium; *n* = 20)
Age, years	70 ± 8	70 ± 7	70 ± 9
Male, no. (%)	23 (57.5)	11 (55)	12 (60)
Etiology of heart failure, no. (%)			
Ischemic heart disease	22 (55)	12 (60)	10 (50)
Hypertensive heart disease	13 (32.5)	6 (30)	7 (45)
Others	5 (12.5)	2 (10)	3 (15)
NYHA class, no. (%)			
I	13 (32.5)	7 (35)	6 (30)
II	27 (67.5)	13 (65)	14 (70)
FEV1.0(%predict)	77.9 ± 5.4	78.1 ± 5.7	77.7 ± 5.7
FEV1.0/FVC, %	59.3 ± 5.0	59.3 ± 5.3	59.2 ± 5.0
Heart rate, beats/min	74.5 ± 5	73 ± 6	76 ± 5
Systolic BP, mmHg	120 ± 7	120 ± 6	119 ± 9
Diastolic BP, mmHg	80 ± 8	79 ± 10	81 ± 7
LVEF, %	36.5 ± 2.0	36.3 ± 2.4	36.6 ± 1.8
BNP, pg/mL	372 ± 105	374 ± 94	369 ± 119
Medications, no. (%)			
Beta-blockers	22 (55)	11 (55)	11 (55)
ACE-I or ARB	40 (100)	20 (100)	20 (100)
CCB	3 (7.5)	1 (5)	2 (10)
Diuretics	30 (75)	16 (80)	14 (70)

NYHA = New York Heart Association, FEV = forced expiratory volume, FVC = forced vital capacity, BP = blood pressure, LVEF = left ventricular ejection fraction, BNP = brain natriuretic peptide, beta-blocker = beta-adrenergic receptor blocker, ACE-I = angiotensin-converting enzyme inhibitor, ARB = angiotensin II receptor blocker, CCB = calcium-channel blocker.

**Table 2:** Group A: tiotropium + observation.

	Day 1	Day 29	Day 56	ANOVA
Systolic BP, mmHg	120 ± 6	115 ± 5 §	118 ± 4 ¶	<0.01
Diastolic BP, mmHg	79 ± 10	75 ± 9 ¶	74 ± 9 §	<0.01
Heart rate, bpm	73 ± 6	66 ± 5 ¶	68 ± 4 ¶	<0.05
BW, kg	59.5 ± 13.7	59.0 ± 13.6	59.3 ± 13.5	NS
SpO_2_, %	96.2 ± 1.7	97.0 ± 1.3 ¶	96.3 ± 1.8	<0.01
Respiratory function				
FEV1.0, L	1.56 ± 0.11	1.74 ± 0.16 §	1.51 ± 0.15	<0.001
FEV1.0(%predict), %	78.1 ± 5.7	87.2 ± 7.9 §	75.7 ± 7.4	<0.001
FVC, L	2.64 ± 0.14	2.75 ± 0.13 §	2.55 ± 0.13	<0.001
FEV/FVC, %	59.3 ± 5.3	63.6 ± 6.4 §	59.5 ± 6.0	<0.001
Echocardiography				
LVDd, mm	57.3 ± 3.7	59.3 ± 3.6 ¶	56.2 ± 3.2	<0.05
LVDs, mm	49.5 ± 66.9	48.3 ± 4.2	48.0 ± 3.6	NS
LVEF, %	36.3 ± 2.4	41.8 ± 5.9 §	37.8 ± 7.8	<0.01
PG(RA-RV), mmHg	18.9 ± 4.8	16.7 ± 4.3 §	16.5 ± 5.1 §	<0.05
IVC, mm	9.7 ± 1.8	9.6 ± 1.7	9.5 ± 1.6	NS
Laboratory testing				
BNP, pg/mL	374 ± 94	263 ± 92 §	293 ± 78	<0.001
Norepinephrine, pg/mL	821 ± 251	468 ± 203 §	501 ± 191 ¶	<0.001

The shaded part of the table indicates the periods of inhalation of tiotropium. § = *p* < 0.01, ¶ = *p* < 0.05 vs. before inhalation, respectively; 6MWD = 6-min walk distance, BP = blood pressure, bpm = beats per minute, BW = body weight, SpO_2_ = arterial oxygen saturation, FEV = forced expiratory volume, FVC = forced vital capacity, LVDd = diastolic left ventricular diameter, LVDs = systolic left ventricular diameter, LVEF = left ventricular ejection fraction, PG(RA-RV) = pressure gradient between the right atrial and right ventricular chamber, IVC = diameter of the inferior vena cava, BNP = brain natriuretic peptide, ANOVA = repeated-measures analysis of variance, NS = no significant change.

**Table 3:** Group B: observation + tiotropium.

	Day 1	Day 29	Day 56	ANOVA
Systolic BP, mmHg	119 ± 9	118 ± 8	113 ± 7 §	<0.01
Diastolic BP, mmHg	81 ± 7	80 ± 7	75 ± 7 ¶	<0.05
Heart rate, bpm	76 ± 5	73 ± 4	68 ± 4 ¶	<0.01
BW, kg	62.1 ± 13.4	61.9 ± 13.5	61.9 ± 13.6	NS
SpO_2_, %	95.6 ± 1.1	96.6 ± 1.1	97.8 ± 0.8	<0.01
Respiratory function				
FEV1.0, L	1.55 ± 0.11	1.60 ± 0.12	1.75 ± 0.18 §	<0.001
FEV1.0(%predict), %	77.7 ± 5.7	80.0 ± 6.0	87.4 ± 8.9 §	<0.001
FVC, L	2.63 ± 0.12	2.64 ± 0.13	2.75 ± 0.11 §	<0.001
FEV/FVC, %	59.2 ± 5.0	60.6 ± 5.0	63.6 ± 5.7 §	<0.001
Echocardiography				
LVDd, mm	57.4 ± 3.1	57.1 ± 3.1	59.0 ± 3.1 ¶	<0.05
LVDs, mm	49.1 ± 6.2	49.5 ± 4.2	48.7 ± 3.8	NS
LVEF, %	36.6 ± 1.8	35.7 ± 3.8	41.6 ± 3.8 §	<0.01
PG(RA-RV), mmHg	19.6 ± 5.1	18.7 ± 5.2	16.5 ± 4.3 ¶	<0.05
IVC, mm	9.5 ± 1.4	9.7 ± 1.6	9.5 ± 2.2	NS
Laboratory testing				
BNP, pg/mL	369 ± 119	358 ± 110	246 ± 101 §	<0.001
Norepinephrine, pg/mL	826 ± 248	747 ± 241	446 ± 107 ¶	<0.001

The shaded part of the table indicates the periods of inhalation of tiotropium. § = *p* < 0.01, ¶ = *p* < 0.05 vs. before inhalation, respectively; 6MWD = 6-min walk distance, BP = blood pressure, bpm = beats per minute, BW = body weight, SpO_2_ = arterial oxygen saturation, FEV = forced expiratory volume, FVC = forced vital capacity, LVDd = diastolic left ventricular diameter, LVDs = systolic left ventricular diameter, LVEF = left ventricular ejection fraction, PG(RA-RV) = pressure gradient between the right atrial and right ventricular chamber, IVC = diameter of the inferior vena cava, BNP = brain natriuretic peptide, ANOVA = repeated-measures analysis of variance, NS = no significant change.
